# Case of Uterine Polypus

**Published:** 1869-02

**Authors:** George W. Hall

**Affiliations:** Carthage, Illinois; Professor of Physiology, Pathology and Therapeutics, Iowa State University


THE
### IOWA MEDICAL JOURNAL.

VOL. V. *January & February*, 1869. No. 4.

### CASE OF UTERINE POLYPUS.

BY GEO. W. HALL, M. D., CARTHAGE, ILLINOIS.

Professor of Physiology, Pathology and Therapeutics, Iowa State University.

Mrs. W., aged 34 years, consulted me in July, 1866. She was the mother of two children, and had generally been healthy until three years before, when she had attacks of profuse bleeding at her menstrual periods. About that time she discovered a tumor of the size of a hen's egg, which she thought was attached to her womb. During the year preceding her visit to me, the tumor had rapidly increased in size, and the bleedings had been more and more frequent and profuse. Upon examination, I found a tumor as large as a child's head at term, completely filling the vagina, passing upward against the bladder, backward against the rectum and resting below on the perineum. The bowels were not often evacuated, and defecation was difficult and extremely painful. She was greatly reduced from the frequent, profuse bleedings. I could not reach the pedicle of the tumor, and could only conjecture its size. I advised ligation and the removal of the tumor as her only safety. To this she consented. Believing that whip cord would not answer for the ligation, some silver wire was procured for that purpose. On the 6th of August, assisted by Dr. Noyes, I attempted to pass the wire around the tumor in the usual manner, with Gooch's double conulæ, but found it impossible, so completely was the vagina filled with the tumor. She was then placed on her knees and elbows; the wire withdrawn from the conula, was looped at its middle over my finger, and pushed up over the tumor posteriorly until it fell back against the pedicle, where it was held while the patient was turned on her back. The ends of the wire were then pulled upward along the sides of the vagina until they met in front, and were then passed through the conulæ, the conulæ pushed up, and the ends of the wire tightened and fastened below in the usual manner. The wire was tightened daily for thirteen days, when the pedicle was cut through and the tumor removed with forceps. The vagina was cleansed daily with injections of solution of permanganate of potassa. Mrs. W. has since menstruated regularly, and has had excellent health. The tumor was of a dense, fibrous structure.

